# A105 PILOT STUDY ON THE ACCURACY OF CHATGPT IN ARTICLE SCREENING FOR SYSTEMATIC REVIEWS IN GASTROENTEROLOGY

**DOI:** 10.1093/jcag/gwad061.105

**Published:** 2024-02-14

**Authors:** C B Na, G Sinanian, N Gimpaya, A Mokhtar, D Chopra, M Scaffidi, E Yeung, S Grover

**Affiliations:** Gastroenterology, St Michael's Hospital, Toronto, ON, Canada; Gastroenterology, St Michael's Hospital, Toronto, ON, Canada; Gastroenterology, St Michael's Hospital, Toronto, ON, Canada; Gastroenterology, St Michael's Hospital, Toronto, ON, Canada; Gastroenterology, St Michael's Hospital, Toronto, ON, Canada; Medicine, Queen's University Faculty of Health Sciences, Kingston, ON, Canada; Scarborough Health Network, Scarborough, ON, Canada; Gastroenterology, St Michael's Hospital, Toronto, ON, Canada

## Abstract

**Background:**

Systematic reviews synthesize extant research to answer a research question in a way that minimizes bias. After articles for potential inclusion are identified by sensitive searches, screening requires human expert review, which may be time-consuming and subjective. Large language models such as ChatGPT may have potential for this application.

**Aims:**

This pilot study aims to assess the accuracy of ChatGPT 3.5 in screening of articles for systematic reviews in gastroenterology by (1) identifying if articles were correctly included and (2) excluding articles reported by authors as difficult to assess.

**Methods:**

We searched the Cochrane Library for gastroenterology systematic reviews (January 1, 2022 to May 31, 2023) and selected the 10 most cited studies. The test set used to determine the accuracy of Open AI’s ChatGPT 3.5 model for included studies was the final list of included studies for each Cochrane review. The test set used for studies challenging to assess was the “excluded studies” list as defined in the Cochrane Handbook. Figure 1 shows the prompt used for the screening query. Articles were omitted if they did not have digital sources, abstracts or methods. Each article was screened 10 times to account for variability within ChatGPT’s outputs. Articles with ≥5 inclusion results were counted as an included study.

**Results:**

ChatGPT correctly identified included studies at rates ranging from 60% to 100%. ChatGPT correctly identified exlcuded studies at rates ranging from 0% to 50% (Table 1). A total of 265 articles were screened.

**Conclusions:**

In this pilot study, we demonstrated that ChatGPT is accurate in identifying articles screened for inclusion in Cochrane reviews; however, it is inaccurate in excluding articles described by the authors as being difficult to assess. We hypothesize that the GPT 3.5 model can read for keywords and broad interventions but is unable to reason cognitively, as an expert would, as to why a study may be excluded. We aim to review reasons for exclusion in future work.

Table 1. Screening Results of ChatGPT

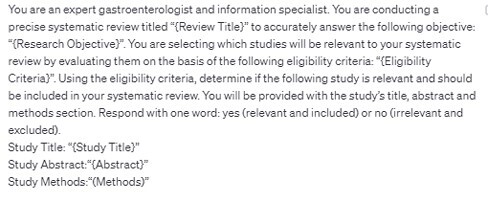

Figure 1. ChatGPT Screening Prompt

**Funding Agencies:**

None

**Table:** 

Review author and date	Topic	No. of studies included by authors	No. of studies excluded by authors	No. of studies correctly included by ChatGPT (%)	No. of studies correctly excluded by ChatGPT(%)
Tse, 2022	Guide-wire assisted cannulation	7	14	7 (100%)	0 (0%)
Gordon, 2023	Remote care through telehealth for IBD patients	14	10	14 (100%)	3 (30%)
Candy, 2022	Mu-opioid antagonists for opioid-induced bowel dysfunction	10	7	10 (100%)	0 (0%)
El-Nakeep, 2022	Stem cell transplantation in Crohn	7	10	7 (100%)	5 (50%)
Okabayashi, 2022	Certolizumab pegol in Crohn	5	5	3 (60%)	1 (20%)
Gordon, 2023	Patient education in IBD management	19	20	18 (95%)	2 (10%)
Dichman, 2022	Antibiotics for uncomplicated diverticulitis	6	6	6 (100%)	0 (0%)
Grobbee, 2022	Faecal occult blood tests versus faecal immunochemical tests for colorectal cancer screening	53	26	46 (87%)	2 (8%)
Midya, 2022	Fundoplication in laparoscopic Heller	9	3	8 (89%)	0 (0%)
Imdad, 2023	Fecal transplantation for IBD	13	21	13 (100%)	2 (10%)

